# Development and Psychometric Properties of a Computer-Based Standardized Emotional Competence Inventory (MeKKi) for Preschoolers and School-Aged Children

**DOI:** 10.1007/s10578-021-01206-6

**Published:** 2021-06-11

**Authors:** Tina In-Albon, Maxim Shafiei, Hanna Christiansen, Tanja Könen, Raphael Gutzweiler, Julian Schmitz

**Affiliations:** 1 Department of Psychology, Clinical Child and Adolescent Psychology and Psychotherapy, University of Koblenz-Landau, Campus Landau, Ostbahnstrasse 12, D-76829 Landau, Germany; 2 Department of Psychology, Clinical Child and Adolescent Psychology, Philipps University Marburg, Marburg, Germany; 3 Department of Psychology, University of Koblenz-Landau, Campus Landau, Landau, Germany; 4 Center for Research On Individual Development and Adaptive Education of Children At Risk (IDeA), Frankfurt, Germany; 5 Department of Clinical Child and Adolescent Psychology, University of Leipzig, Leipzig, Germany; 6 Leipzig Research Center for Early Child Development, Leipzig University, Leipzig, Germany

**Keywords:** Emotional competence, Assessment, Preschoolers, Computer-based measure

## Abstract

A computer-based emotional competence inventory for preschoolers and school-aged children (MeKKi) was developed to assess five components of emotional competence: emotion vocabulary, emotion identification (situational, visual, auditory), emotion understanding, emotion expression, and emotion regulation. Validity, reliability, and factor structure were examined in a community sample of 313 preschoolers and school-aged children (164 boys, 145 girls, 4 n.a.) age 4–11 years (*M* = 6.35 years, SD = 1.85). Item statistics and Cronbach’s α were calculated for the subscales. The unidimensionality of the subscales was additionally tested via item response theory or confirmatory factor analysis. Internal consistency (α) was overall satisfactory at 0.82, though the consistencies of the Visual and Auditory Emotion Identification subscales were lower. Unidimensionality was demonstrated for all subscales except Emotion Understanding. Results provide support for the use of the MeKKi in research and clinical settings to assess emotional competence.

## Introduction

Emotions are part of everyday life. They influence how people think and behave regarding themselves and others. Therefore, people have to be able to deal with, identify correctly, and react appropriately to emotions [1, 2]. According to Denham [2], emotional competence describes the ability of an individual to deal with emotions. It includes emotion expression and experience, emotion understanding of oneself and others, and emotion regulation.

Emotion expression summarizes an individual’s ability to understand the experience and expression of emotions both verbally and nonverbally. Children learn to identify expressed basic emotions within their first year of life, although not yet consciously [3], which then constantly improves. In this process, primary emotions are learned before secondary ones, and positive ones before negative [4]. Emotion understanding refers to knowledge about the emotions of oneself and others and consists of three main components: (a) facial identification of emotions, (b) situational identification of emotions, and (c) vocabulary of emotions [5]. The first describes the knowledge of the different forms of facial expression of emotions. Situational identification refers to the extent to which an individual is capable of connecting situational contexts with the corresponding emotions and vice versa. Finally, making such connections requires an appropriate vocabulary on a verbal and probably also on a conceptual level. Emotion regulation describes the capacity to deal with one’s own negative or positive emotions in a functional way that is both holistic and goal oriented. Children’s successful emotion regulation contributes to their overall emotional competence as it is adaptive to the demands of the context and age-related expectations [6].

Facets of emotional competence are valid predictor of socioemotional well-being, healthy social relationships, academic success, and general physical health [7–10], and their validity is well established. Further, through several studies there is a well-established association of emotional competence with the different components (as described above) and mental health. Difficulties in emotion regulation have been shown to correlate with externalizing [e.g., 10–16] and internalizing [13, 17] symptoms. A meta-analysis by Trentacosta and Fine [18] with 84 studies indicated a relatively consistent yet modest relation between emotion knowledge—a construct similar to emotion understanding—and internalizing and externalizing problems. Emotion knowledge also facilitates emotion regulation [19]. Further, there is an important contribution of emotional competence to academic [20, 21] and social [2, 22] competence. Preschool-aged children’s understanding of emotions often relates to positive peer status, prosocial behaviors, and discrete social behaviors [22–26].

Considering these findings, it is apparent that on the one hand, early deficits in emotional competence are linked to the development of behavior problems and subsequently to the early emergence of mental disorders. On the other hand, emotional competence also facilitates positive social skills and competence [26, 27]. The development of emotional competence begins in early childhood (e.g., sucking a thumb for self-soothing, social referencing when experiencing a current emotion) and continues throughout life, with preschoolers already being adept in some of the components of emotional competence. One study by Saarni [28] found that early childhood (preschool age) is a crucial time for the development of the first stages of emotional competence. This time serves as an early playful testing ground for emotional competence, beginning with successful initiation of a child’s first peer relationships at the age of 2 to 5 years [29].

Assessing emotional competence should be possible already at the preschool age and early school-age. Therefore, assessment measures for preschoolers and school-aged children with sufficient psychometric properties are required. Several instruments have been developed to measure emotional competence in early childhood (e.g., the Assessment of Children’s Emotions Skills [30] and the Diagnostic Analysis of Nonverbal Accuracy [DANVA; 31]), for reviews see McKown et al. [32] or McKown [33]; there is no claim for a complete overview on emotional competence measures. Overall, there are only a few validated instruments for the preschool age (e.g., the Children and Adolescents’ Recognition of Emotions [30], the Emotion Matching Task [34], and the Affect Knowledge Test shortened [AKT-S, 35]), and these instruments are available only in English, indicating the necessity of a German instrument. There is also a lack of computerized tests, one example is the computerized adaptation of the AKT-S [36]. Overall, there is a lack of validated computer-based preschool emotional competence instruments in German. The need for an additional instrument was also indicated by child psychotherapists who participated in an expert rating in a pilot study of the MeKKi (see below). Existing measures in German predominantly assess singular components of emotional competence [37], such as the ET 6-6R, a development test for ages 6 months to 6 years [38, 39] that assesses general emotional development; the Scale of Emotion Knowledge for 3- to 10-Year-Old Children [40]; and the Questionnaire on Strategies of Anger Regulation for Children [41]. The FEEL-KJ [42] is a self-report questionnaire that assesses emotion regulation strategies (adaptive and maladaptive) for the emotions anxiety, sadness, and anger. In conclusion, as outlined above, emotional competence consists of several components that should already be directly assessable in preschoolers and not only with caregivers. Therefore, to assess several components of emotional competence in preschoolers and school-aged children, a new computer-based standardized measure, the Emotional Competence Inventory for Children (referred to here by its German acronym, MeKKi), was developed; the measure is based on Denham’s [2] model of emotional competence and Ekman´s definition of emotions [43, 44]. We investigated the psychometric properties (reliability and validity) and factor structure of the MeKKi in a preschooler and school-aged sample.

## The MeKKi: A New Measure of Emotional Competence

### Development of the MeKKi

The MeKKi was developed as a computer-based measure assessing several components of emotional competence in children between 4 and 10 years of age [45]. The theoretical basis of the MeKKi is Denham’s [2] multidimensional model of emotional competence, as outlined above, and the basic emotions from Ekman and Friesen [43, 44]. Following Denham’s model, the MeKKi consists of the following subscales: Emotion Expression, Emotion Understanding, Emotion Vocabulary, Emotion Identification (Situational, Visual, and Auditory), and Emotion Regulation. The Emotion Vocabulary and Emotion Identification subscales map to Denham’s emotion understanding component.

### The MeKKi Subscales

#### Emotion Expression

This subscale assesses the child’s ability to express facial emotions such as happiness, sadness, fear, and anger and a neutral facial expression. The neutral facial expression has been included for exploratory reasons for further studies on cognitive biases. The child is asked to twice visually model 10 emotions with their own facial expression. The researcher then judges the shown expression on a 4-point Likert scale (0 = no correspondence, 1 = slightly corresponds, 2 = corresponds, 3 = corresponds exactly). The researcher has exemplary children’s pictures for reference, taken from an evaluated database (Radboud Faces Database [46]). These pictures are displayed in the test manual together with the most important factors of facial expression according to Ekman [47]. Interrater reliability (Cohen’s kappa) of two independent researchers of the total sample was 0.66. Results did not change, whether neutral facial expressions were included or not.

#### Emotion Understanding

This subscale assesses the child’s ability to differentiate between emotions. The researcher refers to 13 emotions and emotion-related constructs (happiness, fear, sadness, anger, disgust, surprise, love, sympathy, pride, disappointment, shame, guilt, jealousy). The child has to give an example of a situation from their own life when they felt this way. The researcher rates on a 4-point Likert scale (0 = no correspondence, 1 = slightly corresponds, 2 = corresponds, 3 = corresponds exactly) whether the described situation corresponds to the emotion. If the child describes an event that also fits another emotion, this is accounted for in the scoring as 1, following Harris et al. [48]. Intraclass correlation for this subscale ranged from 0.66 to 1.00 for the different emotions.

#### Emotion Vocabulary

This subscale assesses how many emotional words the child knows. The child is given an example of an emotion and then asked to mention all the emotion words they know. The interrater reliability of this subscale was 0.92.

#### Emotion Identification

For data assessment, emotion identification is based on the three subscales Situational, Visual, and Auditory Emotion Identification, although intercorrelations between these subscales are expected.

##### Situational Emotion Identification

This subscale assesses the child’s ability to identify their own emotions in different situations. Emotion identification is part of emotion understanding in Denham’s model, including that a child is able to assign situations to emotions. As for the emotion expression interview [49], situations are chosen to correspond to the emotions happiness, sadness, anxiety, anger, surprise, and disgust as well as a neutral non-emotion arousing situation. The child is presented with 12 different vignettes of emotional situations (e.g., “It’s your birthday and you are celebrating with your friends. There is a cake, games, and presents.”). Most of the vignettes were translated from Ribordy et al. [50]. New vignettes were developed for anxiety and neutral. To assess emotion identification, the child is asked which emotion they would have in the given situation. The answer options are happiness, sadness, fear, anger, and a neutral non-emotion arousing state. There were two vignettes for each emotion and two vignettes for mixed emotions (sadness and anger; sadness and anxiety).

##### Visual Emotion Identification

This subscale assesses the child’s ability to identify other people’s emotional states in emotional facial expressions. The child is shown 26 facial expressions of different people (girls, boys, women, men) on facial images (Radbound Faces Database) [46] presented on a screen, each for approximately 5 s. The child has to name the expression with one of Ekman’s [47] six basic emotions (happiness, anger, sadness, fear, disgust, surprise) or an additional neutral expression. For the pictures (and the auditory files of the Auditory Emotion Identification subscale), pretests were conducted for feasibility and understanding. The visual and auditory tasks are similar to those used in the DANVA [31].

##### Auditory Emotion Identification

The child is presented with 10 auditory stimuli that consist of a spoken sentence (“I did that.”) that is always the same but with varying emotional intonations. The emotional intonations are sadness, fear, anger, happiness as well as a neutral non-emotional intonation. The procedure was according to Nowicki and Duke [31]. The child is asked to identify the emotion of the speaker of the auditory stimuli.

#### Emotion Regulation

The child listens to six vignettes that could induce negative emotions (sadness, anger, fear each twice). The child is asked what strategies they know to regulate negative emotions, such as sadness, anger, and fear. Thus, the subscale assesses knowledge of strategies to regulate emotions. The child is allowed to name two strategies, which are then categorized as adaptive (functional) or maladaptive (dysfunctional) according to Saarni [22, 51] or Grob and Smolenski [42] by the interviewer following the manual that includes several examples, such as accepting, problem solving, or reappraisal for adaptive strategies and perseveration, withdrawal, and resignation for maladaptive strategies. The interrater reliability of the total sample for the different strategies for each emotion ranged from 0.63 to 0.85.

### Pilot Studies on the Psychometric Properties of the MeKKi

#### Pilot Study 1: Initial Examination of Feasibility Using an Expert Rating.

In the first pilot study, the feasibility and external criterion validity of the MeKKi were investigated in an expert sample of 42 child and adolescent psychologists and psychotherapists using school grades (i.e., 1 = excellent, 6 = insufficient). They received a written description of the MeKKi with the intended age from 5 to 9 years of age and then administered the measure. Eighty percent of the experts rated the MeKKi as good or very good regarding age adequacy, design, implementation, and quality. The median of the overall rating was 2, indicating a good grade.

#### Pilot Study 2: Examination of Measure Acceptability

In the second pilot study, a community sample of 75 children between 4 and 9 years of age (*M* = 7.1 years, SD = 1.5) completed the MeKKi and rated their acceptance of it. The majority of the children stated that they liked the MeKKi (92%), had enjoyed taking the test (87%), and had understood how to take it (83%).

#### Pilot Study 3: Examination of Differential and Construct Validity

In the third pilot study, for differential validity of the MeKKi, school children (*n* = 46; *M*_age_ = 6.91 years, SD = 1.62) were compared with children in the youth welfare system (*n* = 33; *M*_age_ = 7.30 years, SD = 1.29), that is, a group at risk for psychological problems [52] and emotion regulation difficulties [53, 54]. Results indicate that the MeKKi differentiated between the two groups across most subscales, with the at-risk sample showing lower emotional competence. Significant group differences were found in Emotion Vocabulary (*d* = 0.51), Situational Emotion Identification (*d* = 0.66), and Emotion Understanding (*d* = 0.76), controlled for verbal ability. For the Visual and Auditory Emotion Identification subscales, the means for the children in the youth welfare system were lower than those for the school children, but these differences were not significant. The results of the Emotion Regulation subscale were similar, with fewer adaptive and more maladaptive strategies found for the children in the youth welfare system.

For construct validity, there were significant positive intercorrelations of the MeKKi subscales. Criterion validity was assessed with the correlation of the MeKKi subscales with the Strengths and Difficulties Questionnaire (SDQ). Internal consistency (Cronbach’s α) of the total score of the SDQ was 0.82. The correlations were not significant. However, the correlations were in the expected directions; that is, positive correlations with prosocial behavior and negative correlations of the MeKKi subscales with problem behaviors were found.

#### Pilot Study Discussion

The results from these three pilot studies provided some preliminary evidence of the feasibility, acceptability, and validity of the MeKKi. Nevertheless, the small sample size has to be considered as an explanation of the lack of significance of the correlations with other measures. The results also indicated that some changes to the first version of the MeKKi were necessary (e.g., including examples for the tasks, revising auditory vignettes in the Emotion Identification subscale for anxiety and sadness, adding disgust and surprise to the Visual Emotion Identification subscale, and adding secondary emotions such as pride, empathy, and jealousy [43] to the Emotion Understanding subscale because of ceiling effects with primary emotions).

## Study 1

The goal of Study 1 was to investigate the revised version of the MeKKi, especially the interrater reliability, retest reliability, and internal consistencies.

### Method

The sample consisted of 45 children between 5 and 11 years of age (*M* = 7.44 years, SD = 1.71, 22 girls, 23 boys), recruited in schools and kindergarten. Participants and their parents provided written informed consent and the children were each investigated in a one-on-one setting.

### Results

The Emotion Vocabulary subscale, which includes open questions, had a very high interrater reliability of 0.99 between independent researchers. The interrater reliability was 0.66 for Emotion Expression, 0.85 for Emotion Regulation, and 0.71 for Emotion Understanding. The 7-week retest reliability was *r* = 0.40 and *r* = 0.46 in a small subsample of *n* = 13 for the Emotion Vocabulary and Emotion Identification (visual) subscales, respectively. The other subscales and the total score resulted in larger correlations, ranging from *r* = 0.59 Emotion Identification (auditive), r = 0.71 for Emotion Understanding, r = 0.86 for Emotion Identification (situational), r = 0.81 for Emotion Regulation to *r* = 0.88 (Emotion Understanding). Positive correlations were found between nearly all subscales to varying degrees. Cronbach’s α for Emotion Identification was 0.57, for Emotion Expression 0.75, for Emotion Regulation 0.79, and for Emotion Understanding 0.76. Considering these results, a larger sample of preschoolers and school-aged children was recruited, with which we used for Study 2.

## Study 2

### Method

#### Participants

A total of 313 children (164 boys, 145 girls, 4 n.a.) between the ages of 4 and 11 years (*M* = 6.35 years, SD = 1.85; boys: *M* = 6.55 years, SD = 1.86; girls: *M* = 6.10 years, SD = 1.80) participated in the study. For data analysis, the sample was split into two age groups: One group, called preschoolers, consisted of 151 children (70 boys, 81 girls) below the age of 7 years (*M* = 4.81 years, SD = 0.77); the other group (school-aged) consisted of 136 children (81 boys, 54 girls, 1 n.a.; the age was missing for 26 children, but the grade of these children was known) aged 7 years and older (*M* = 8.06 years, SD = 0.99). Testing these two age groups served as a cross-sectional design and allowed us to test the external validity by widening the age range of the MeKKi from preschoolers to early-school-aged children, to obtain preliminary reliability results for a larger age-range of children. The age cut-off was based on the German education system, in which children typically start school at around 6 years of age (including children up to 6 years and 11 months). The children were recruited from preschools and primary schools at three German sites, Landau (*N* = 73), Leipzig (*N* = 107), and Marburg (*N* = 133). Not all study sites conducted all subscales of the MeKKi. Therefore, there are different sample sizes for the subscales, as indicated in Table 1.

**Table 1:** Means and standard deviations by age (preschoolers vs. school-aged children), Welch test, and effect sizes for testing mean differences between age groups

Subscale	Preschoolers			School-aged children			*t*_w_	*df*	*p*	*d*
	*n*	*M*	SD	*n*	*M*	*SD*				
EE (27)	65	16.69	6.22	132	18.57	5.73	2.04	118.48	0.043	0.31
EI-A (10)	67	5.82	1.88	136	7.35	1.72	5.60	121.64	< 0.001	0.86
EI-S (13)	151	5.89	3.02	135	9.41	1.55	12.57	229.11	< 0.001	1.44
EI-V (26)	80	16.94	3.81	136	19.32	2.82	4.86	130.29	< 0.001	0.74
ER (32)	151	12.79	7.05	135	22.01	6.09	11.87	283.66	< 0.001	1.39
EU (39)	48	20.25	8.21	82	30.22	6.67	7.14	83.18	< 0.001	1.37

*EE* emotional expression, *EI* emotion identification, *A* auditory, *S* situational, *V* visual, *ER* emotion regulation, *EU* emotion understanding; maximum reachable scores of subscales shown in parentheses; *t*_w_ = tested value of *t* test with Welch correction; *d* = Cohen’s *d*. Preschoolers were under 7 years old; school-aged children were 7 years old or above

#### Procedure

Parents provided written informed consent and the children provided informed assent to participate in this study. The study was approved by the ethics committee of the Department of Psychology of the University of Koblenz-Landau. The children completed the test in a one-on-one setting in a separate quiet room on a comfortable chair in good view of a computer screen, on which the MeKKi was presented. During the assessment a researcher was present who was diagnostically trained, was under supervision, and was given a test manual (including examples and facial pictures) for the assessment of the MeKKi. Further, the researchers first observed assessments before conducting assessments themselves. Items or instructions were read aloud to children who experienced problems reading the items or the instructions. The testing took about 30 min per child, for both the school-aged and the preschoolers. Each subscale of the MeKKi was scored separately.

#### The Measure

The MeKKi is a computer-based standardized measure assessing several components of emotional competence, based on Denham’s model of emotional competence [2] and Ekman’s basic and secondary emotions [43, 44] in preschoolers and school-aged children.

### Data Analysis and Preparation

The characteristic values of all MeKKi subscales were analyzed on both the item and the subscale level. The distributions of all items were calculated together with the difficulty and selectivity of each item. Furthermore, Cronbach’s α was calculated on the subscale level for internal consistency. Item response theory (IRT) was used to test the dimensionality of the subscales utilizing dichotomous (right vs. wrong) items (i.e., the three subscales concerning emotion identification). Both a Rasch model and a Birnbaum model were used. The former hypothesizes that the discriminatory parameters are equal for all items, with difficulty being the sole discriminator. The latter freely estimates for each item its own discriminatory parameter. A parametric bootstrap approach was used to test absolute model fit, then a likelihood ratio test to compare the two models. If a significant difference was found, the less restrictive model was chosen. Relative model fit was also calculated using the Akaike information criterion (AIC) and the Bayesian information criterion (BIC). These have in common that lower values indicate better model fit. Additionally, the Anderson likelihood ratio test [55] was used to test the goodness of fit within age groups for each Rasch model. Since the design of the items prevents a correct estimation of the likelihood of guessing the correct answer, we did not model this parameter through IRT. The Emotion Understanding, Emotion Regulation, and Emotion Expression subscales were tested for their dimensionality through confirmatory measuring models of classic test theory. For each subscale, two models were formed, the first a tau-congeneric model, where all parameters are estimated freely, and the second essentially a tau-equivalent model, where all loading parameters are set equal.

The Emotion Regulation and Emotion Expression subscales consist of multiple items targeting the same emotion. The Emotion Regulation subscale consists of four items for the emotions anger and fear, eight items for the emotion sadness, and two items, one for each of two mixed emotions (sadness–anger and sadness–fear). For each emotion, all items were aggregated because of high intercorrelations between the items. The mixed emotions were not used in the model because of high correlations with the single emotion component. The final model for emotion regulation consisted of three items as indicators (anger, fear, sadness). The Emotion Expression subscale consists of two items for each emotion (except happiness with only one item). Again, the two items for one emotion were aggregated. The final model for emotion expression consisted of the mean value of five emotions. The model of emotion understanding consisted of 13 items. Further, both confirmatory factor analysis (CFA) models were estimated. Measurement invariance was tested between the two age groups, preschoolers and school-aged children, for the Emotion Expression and Emotion Regulation subscales. To inspect model fit, the χ^2^ test and additionally the comparative fit index (CFI), the root mean square error of approximation (RMSEA), and the square root mean residual (SRMR) were used, with CFI values greater than 0.95, RMSEA values smaller than 0.06, and SRMR values smaller than 0.08 indicating acceptable cutoffs [56]. The AIC and BIC were calculated for both models, emotion expression and emotion understanding. Robust maximum likelihood estimation with robust Huber–White standard errors was used to test the models for emotion regulation and emotion expression. To estimate the model of emotion understanding, the weighted least square mean and variance adjusted estimators was used because of the Likert-type scale of the items.

## Results

### Quality Criteria

The objectivity of the MeKKi is given, as all instructions are standardized and provided on a computer screen. The test manual provides standardized instructions for the assessment as well as the ratings of the measure. Reliability is overall satisfactory with the majority of subscales demonstrating acceptable to good Cronbach’s α values (see Table 2). Only for the Visual and Auditory Emotion Identification subscales were Cronbach’s α values below 0.70.

**Table 2:** Means, standard deviations, Cronbach’s α reliability coefficients, and range of item–total correlation for the MeKKi subscales

Subscale	*n*	Scale values				α	*r*_it_	
		*M*	SD	Min	Max		LL	HL
EE (27)	200	18.04	5.95	0	27	0.76	0.28	0.63
EI-A (10)	206	6.88	1.93	1	10	0.55	0.10	0.35
EI-S (13)	312	7.36	3.05	0	11	0.83	0.33	0.75
EI-V (26)	219	18.44	3.40	1	25	0.63	0.01	0.41
ER (32)	312	16.67	8.15	0	32	0.88	0.42	0.62
EU (39)	133	26.77	8.75	0	39	0.81	0.38	0.53

MeKKi is the German acronym for the Emotional Competence Inventory for Children. *EE* emotional expression, *EI* emotion identification, *A* auditory, *S* situational, *V* Visual, *ER* emotion regulation, *EU* emotion understanding; maximum reachable scores of subscales shown in parentheses; *r*_it_ = part-whole-corrected item–total correlation; *LL* lowest measured item value; *HL* highest measured item value

### Measurement Models for Emotion Expression, Emotion Understanding, and Emotion Regulation

All three CFA models demonstrated good model fit for Emotion Expression, Emotion Understanding, and Emotion Regulation (Table 3). The Emotion Expression and Emotion Understanding subscales passed the congeneric scale level and should thus be viewed as unidimensional. Restrictions due to equalizing factor loadings, mean values, or residual variance led in both models to a significant deterioration of model fit. The Emotion Regulation subscale passed the essentially tau-equivalent measurement model level and is therefore unidimensional.

**Table 3:** Model fit of final measurement model for emotion expression, emotion understanding, and emotion regulation

Subscale	χ^2^	*df*	*p*	CFI	RMSEA	*p*	SRMR
Emotion expression	5.30	5	0.381	0.997	0.017	0.636	0.027
Emotion understanding	74.39	65	0.199	0.960	0.033	0.790	0.067
Emotion regulation	1.06	2	0.588	1.00	< 0.001	0.724	0.017

*CFI* comparative fit index, *RMSEA* root mean square error of approximation, *SRMR* square root mean residual. Estimator for emotion expression and emotion regulation was maximum-likelihood with Huber–White correction; the estimator for emotion understanding was the weighted least square mean and variance adjusted estimator. The sample size only takes children into account for whom all items had corresponding values and given age value. Sample size for emotion expression: *n* = 197, for emotion understanding: *n* = 130, and for emotion regulation: *n* = 287

**Fig. 1:**
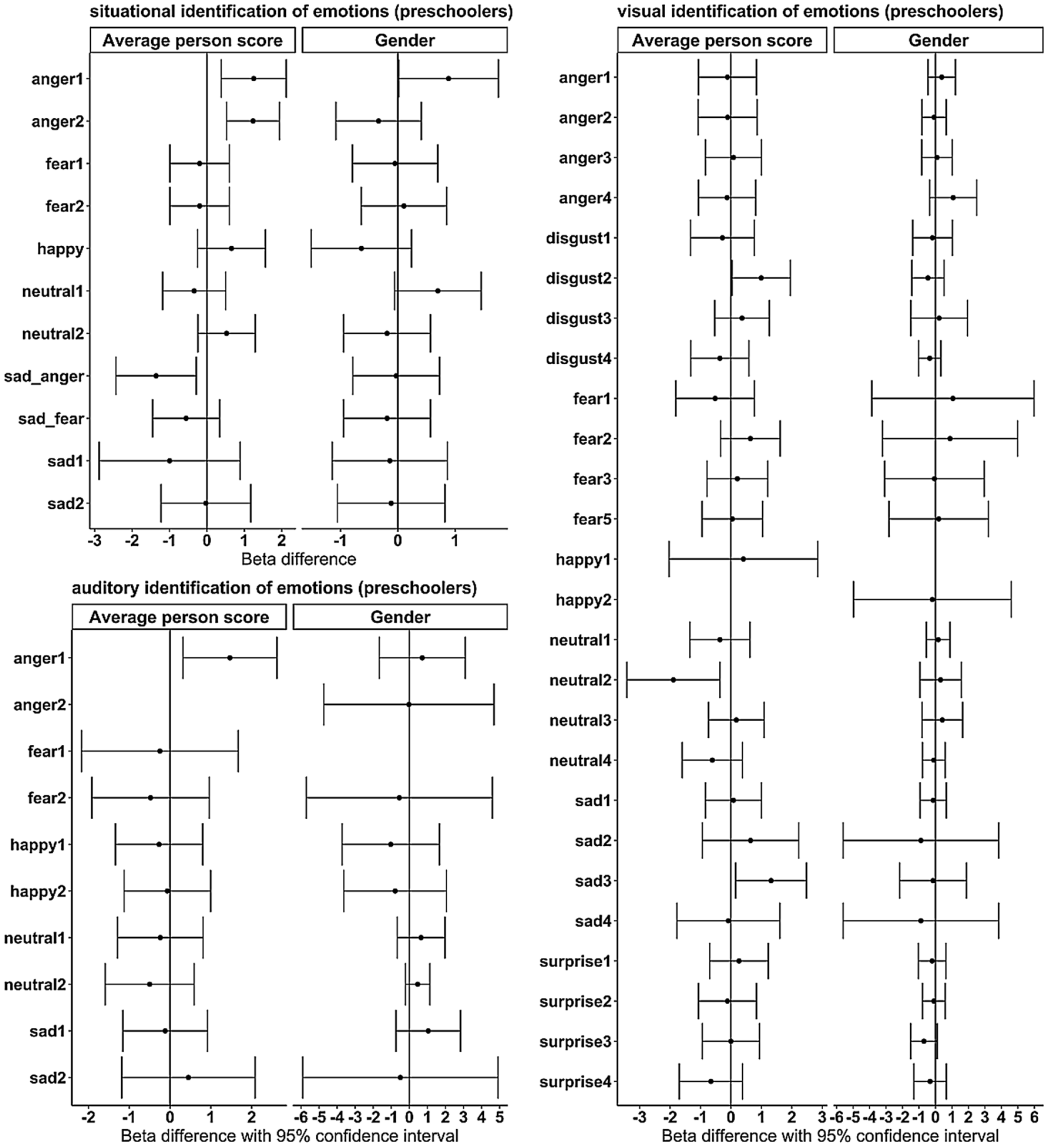
Graphical test of differential item functioning of the situational, auditory, and visual emotion identification of preschoolers (< 7 years old). Plotted are the beta differences for each item. The difference for person score was calculated between betas for children with person scores below and above the average. The difference between genders was calculated between betas for girls and betas for boys; negative values indicate higher difficulty for girls, and positive values indicate higher difficulty for boys. Items have numbers if there were multiple items for an emotion

**Fig. 2:**
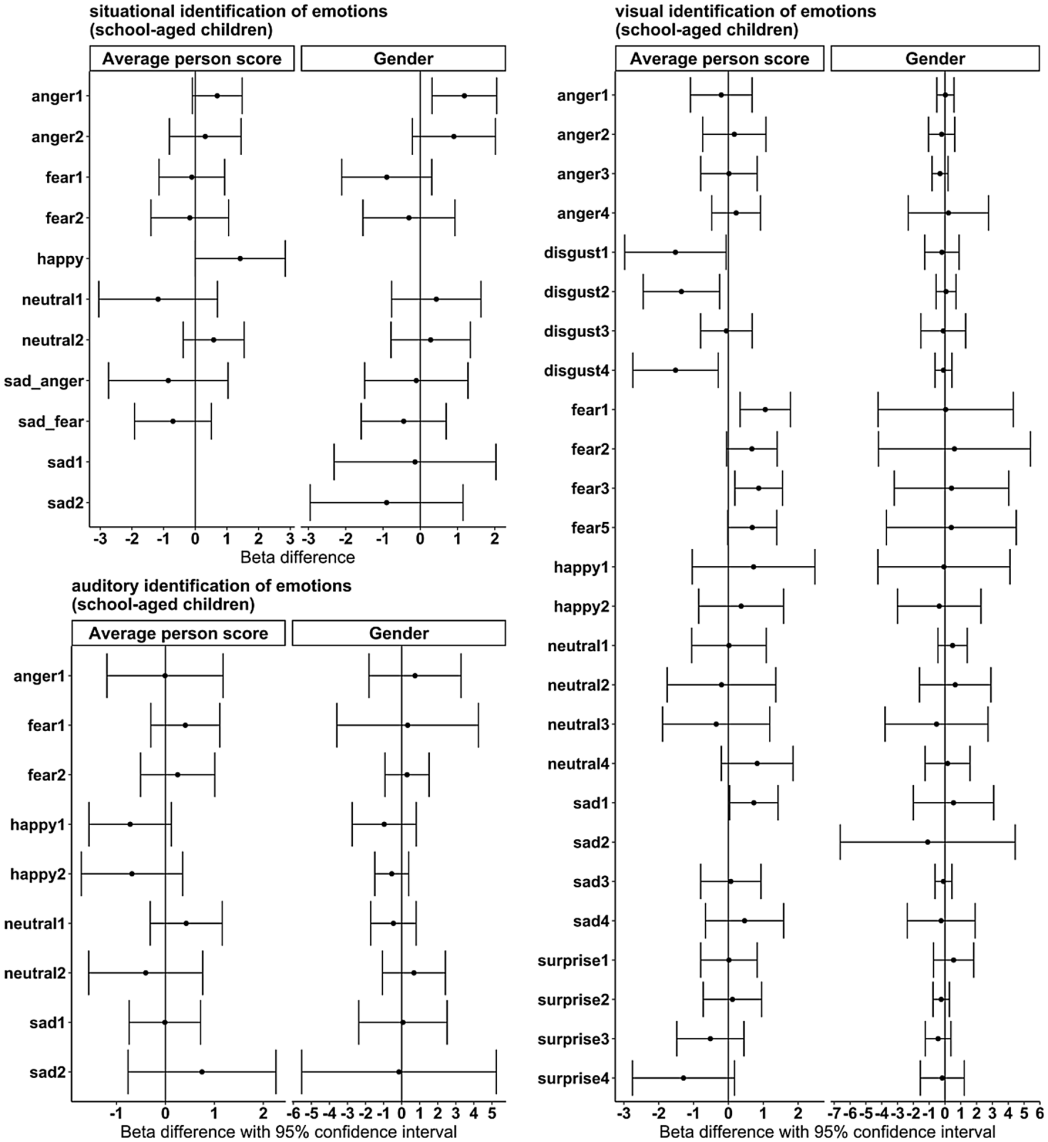
Graphical test of differential item functioning for school-aged children (≥ 7 years old). Plotted are the beta differences for each item. The difference for person score was calculated between betas for children with person scores below and above the average. The difference between genders was calculated between betas for girls and betas for boys; negative values indicate higher difficulty for girls, and positive values indicate higher difficulty for boys. Items have numbers if there were multiple items for an emotion

In terms of measurement invariance, which was tested for differences in the average person score as well as for gender differences (see Table 4), Emotion Expression showed configural measurement invariance for the average person score, χ^2^ = 14.11, *df* = 10, *p* = 0.168; CFI = 0.97, RMSEA = 0.07, SRMR = 0.04 and also for gender, χ^2^ = 13.80, *df* = 10, *p* = 0.182; CFI = 0.98, RMSEA = 0.06, SRMR = 0.04. Probably due to the small sample size, there were some items that could not be considered because of missing variance (see Figs. 1 and 2). Testing for weak measurement invariance led to a somewhat decreased model fit for both grouping variables (ΔCFI > -0.01). The assumption of equal factor loadings therefore had to be dismissed [57, 58]. Testing measurement invariance for Emotion Regulation resulted in strict invariance between the two age groups, ΔCFI < -0.001, χ^2^ = 4.54, *df* = 7, *p* = 0.716; CFI = 1.00, RMSEA < 0.01, SRMR = 0.04 for average person score, and strong invariance, ΔCFI < -0.001, χ^2^ = 7.53, *df* = 4, *p* = 0.111; CFI = 0.991, RMSEA = 0.08, SRMR = 0.05 for gender. Because of the relatively small size of the sample compared to the large number of indicators, measurement invariance could not be tested for Emotion Understanding.

**Table 4:** Results of the model comparison of measurement invariance testing between age groups (preschooler/school-aged children) for Emotion Expression (EE) and Emotion Regulation (ER)

Subscale	Group by	Model	CFI	ΔCFI	Δχ^2^	*df*	*p*	AIC	BIC
EE	Age	Configural	0.966					2625.02	2723.52
		Weak	0.933	− 0.033	7.83	4	0.098	2624.91	2710.28
		Strong	0.905	− 0.028	7.38	4	0.117	2623.96	2696.19
		Strict	0.919	0.014	4.76	5	0.443	2621.18	2677.00
	Gender	Configural	0.968					2598.65	2697.30
		Weak	0.978	0.010	.339	4	0.495	2596.05	2680.55
		Strong	0.881	− 0.111	15.89	4	0.003	2602.12	2674.46
		Strict	0.706	− 0.175	18.62	5	0.002	2632.05	2687.95
ER	Age	Configural	1.000					933.66	999.53
		Weak	1.000	< − 0.001	0.203	2	0.904	930.73	989.28
		Strong	1.000	< − 0.001	0.05	2	0.974	926.79	978.03
		Strict	1.000	< − 0.001	3.57	3	0.312	924.49	964.74
	Gender	Configural	1.000					1043.79	1109.72
		Weak	0.992	− 0.008				1044.10	1102.71
		Strong	0.991	− 0.001	2.70	2	0.259	1042.85	1094.13
		Strict	0.989	− 0.002	3.49	3	0.322	1040.29	1080.59

*AIC* Akaike information criterion, *BIC* Bayesian information criterion, *CFI* comparative fit index, *ΔCFI* difference of CFI compared to previous less restricted model. Difference greater than 0.01 must be marked as problematic [56, 57]. Δχ^2^ = Test value in the χ^2^ difference test. ER consists of only three items, so there was a saturated model for configural invariance and no model testing was possible

### IRT Models for Emotion Identification

Rasch homogeneity could not be reached for the Emotion Identification subscales, although total model fit was partially achieved (see Table 5). The graphical model testing and the likelihood ratio testing according to Anderson [55] showed differential item functioning between the age groups, meaning that item difficulty differed according to age. Therefore, the Emotion Identification subscales were analyzed separately for preschoolers and school-aged children (see Table 5).

**Table 5:** Results of item response theory tests of Emotion Identification (Situational, Visual, Auditory) for preschoolers and school-aged children

Model fit	Preschoolers			School-aged children		
	Situational	Visual	Auditory	Situational	Visual	Auditory
Rasch (1PL)						
Absolute fit	*p* = 0.57	*p* = 0.05	*p* = 0.73	*p* = 0.01	*p* = 0.02	*p* = 0.03
AIC	1831.21	2352.97	742.86	1070.71	3442.12	1400.39
BIC	1864.40	2414.90	764.91	1,02.59	3517.85	1429.52
Birnbaum (2PL)						
Absolute fit	*p* = 0.10	a	*p* = 0.86	*p* = 0.25	a	*p* = 0.02
AIC	1753.89	2274.93	745.69	1063.88	3321.89	1405.88
BIC	1820.28	2398.80	789.78	1127.63	3473.35	1464.14
Model comparison						
χ^2^	99.32	130.04	17.17	28.83	172.22	14.51
*df*	11	26	10	11	26	10
*p* value	< 0.001	< 0.001	0.071	0.002	0.001	0.151

*p p* value of the parametric bootstrap (B = 1000) for absolute model fit. Results of the Rasch model (1PL = one-parameter logistic model) and the Birnbaum model (2PL = two-parameter logistic model) for the Emotion Identification subscales. *AIC* Akaike information criterion, *BIC* bayesian information criterion, *a* Estimation did not converge successfully

The Birnbaum model for situational emotion identification of preschoolers showed a better fit than the Rasch model, χ^2^(11) = 99.32, *p* < 0.001, although the Rasch model achieved a good total model fit as well. The Anderson test showed differential item functioning on the average person score for the items concerning anger between participants above and below the mean sum score and no differential item functioning for gender (see Fig. 1). The Rasch model for visual emotion identification of preschoolers showed a significantly poorer fit for the given data, χ^2^(26) = 130.04, *p* < 0.001. The graphical model test showed differential item functioning on the average person score for two items (one for sadness and one for neutral) and no differential item functioning for gender. There was no significant difference between the Rasch and Birnbaum models for auditory emotion identification of preschoolers, χ^2^(10) = 17.17, *p* = 0.071. One anger item was found to have differential item functioning for participants based on their average person score and no differential item functioning for gender.

For situational emotion identification of school-aged children, the Birnbaum model reached a significantly better fit compared to the Rasch model with the given data, χ^2^(11) = 28.83, *p* = 0.002. Again, the Birnbaum model had a better fit for visual emotion identification, χ^2^(26) = 172.22, *p* < 0.001. Several items showed differential item functioning on the average person score and no differential item functioning for gender (see Fig. 2), in the case of differentiation above and below the mean sum score. For auditory emotion identification, Rasch homogeneity was shown, with the model indicating a good fit for the given data, χ^2^(10) = 14.51, *p* = 0.151. There was no differential item functioning for either grouping variable.

### Intercorrelations

The intercorrelations of the subscales are presented in Table 6. While for preschoolers the correlations between the subscales ranged between low and high (− 0.02 ≤ *r* ≤ 0.65), the range of correlations of the subscales for school-aged children was between low and medium (0.05 ≤ *r* ≤ 0.38). Overall, there were weak to strong correlations between the different subscales for preschoolers and school-aged children. The only negative correlation was between Emotion Expression and Auditory Emotion Identification for preschoolers, but it was not significant, *r* = − 0.02, *p* = 0.855. All other correlations were positive as expected.

**Table 6:** Spearman correlation between MeKKi subscales for preschool children (below diagonal) and school-aged children (above diagonal)

	1	2	3	4	5	6
1 EE	–	0.07	0.38*	0.16	0.22*	0.37*
2 EI-A	− 0.02	–	0.22*	0.22*	0.05	0.11
3 EI-S	0.39*	0.49*	–	0.16	0.35*	0.25*
4 EI-V	0.11	0.38*	0.52*	–	0.08	0.31*
5 ER	0.12	0.04	0.65*	0.27*	–	0.37*
6 EU	0.16	0.10	0.30*	0.29	0.60*	–

*EE* emotion expression, *EI* emotion identification [A = Auditory, S = Situational, V = Visual], *ER* emotion regulation, *EU* emotion understanding. Sample sizes for correlations for preschool children between *n* = 48 and *n* = 151. Sample sizes for correlations for school children between *n* = 82 and *n* = 136; *p < 0.05

## Discussion

The MeKKi was developed to assess different components of emotional competence, that is, emotion vocabulary, emotion understanding, emotion expression, emotion identification (situational, visual, auditory), and emotion regulation, in preschoolers and school-aged children. This is the first German-language instrument that assesses different components of emotional competence and that can be used in preschoolers and school-aged children. Our investigation of the psychometric properties and factor structure of the MeKKi indicates that most of the MeKKi subscales were unidimensional with one latent dimension underlying each subscale. The only exception was Visual Emotion Identification, which showed some estimation problems that need further investigation. Estimates for Cronbach’s α and item–total correlations were in an acceptable to good range for most of the subscales, again with the exception of the Visual Emotion Identification and Auditory Emotion Identification subscales.

There was a wide range of correlations between the subscales, confirming that each scale represents a separate factor of emotional competence. Configural invariance was found between preschoolers (< 7 years) and school-aged children (≥ 7 years) for Emotion Expression and Emotion Regulation, which indicates that the unidimensional structure holds for both age groups. Furthermore, there was strict invariance for Emotion Regulation. Because the sample size was too small, a test of measurement invariance for Emotion Understanding was not possible and has to be addressed in future studies. Unidimensionality was also found for Situational and Auditory Emotion Identification, but only Auditory Emotion Identification reached Rasch homogeneity, while some items of the Situational subscale showed differential item functioning. The Birnbaum model for Visual Emotion Identification for both age groups did not converge successfully. An inspection of the factor scores showed that for most of the factors only one observation was found and the expected value was zero. One problem could be that with increasing item number, an increase of sample size would be required [59]. For Birnbaum models, a sample size of 200 is recommended [60], which was not available for all subscales. Therefore, the combination of a relatively small sample size and the high number of items could be the reason that the model did not converge.

The results add to the previous pilot results of the MeKKi, as presented in the Introduction section, indicating acceptable to good retest reliabilities for the majority of the subscales and high acceptance ratings of the participating children. Examining gender differences, no differential item functioning was shown for any tested subscale and strong invariance was found for Emotion Regulation. The only subscale that showed no invariance was Emotion Expression. On a subscale level, there were no significant group differences between girls and boys, which is in line with other studies on gender differences in emotional competence during early childhood [e.g., 61–63]. Given the very small number of group members, the issue of gender differences must be addressed in further research.

Despite these positive results, there were some problematic items identified in the MeKKi. Several items concerning anger showed differential item functioning for the Situational and Auditory Emotion Identification subscales for preschoolers. This is a known problem, because anger is difficult to distinguish from some other emotions, such as grief [64, 65]. The relatively wide range in ages within this sample could be an explanation for the differential item functioning mentioned above. Another explanation could be that different facial emotion expressions are learned in different life stages. For example, Durand et al. [66] showed that happiness and sadness are recognized earlier than anger or disgust. However, it has to be investigated, if this result is also shown in auditory emotion identification of anger compared to other emotions. Results in the present study indicate that preschoolers had more difficulties with the task than the school-aged children. We also found that children with above-average person scores (indicating higher emotional competence in this study) had a higher probability of answering correctly than children with below-average person scores.

### Limitations

This study has some limitations that have to be acknowledged when interpreting the results. Splitting the sample into two age groups (preschoolers, aged < 7 years, and school-aged children, aged ≥ 7 years) was a reasonable way to address the fact that the development of emotional competence changes with increasing age. It was expected that these differences would reduce the fit of the models. It is possible that there are other differences between the age groups that are not represented by these two groups. However, given the overall sample size, splitting the sample into more age groups was not possible. Further studies should examine the MeKKi for different age groups or should reduce the variance of age within each group to control for age as a variable. Splitting the sample into several age groups, at least for children below the age of 9 years, could be reasonable for future studies given the findings of de Sonneville et al. [67], which indicate that emotional competence stabilizes around age 9 with only a small qualitative increase to be expected afterward. The sample sizes differed in the subscales, which means that scales with fewer tested children were less accurately estimated. Measurement invariance was tested only between groups and not over time, as no repeated measurement data were available. This has to be done in future studies to ensure that the number of dimensions does not change over time and that intercorrelations between items and their respective dimensions remain relatively constant. Like other self-report measures on emotion regulation, the MeKKi assesses knowledge of emotion regulation strategies, as opposed to actual behavioral emotion regulation in different emotional situations. This could be investigated by considering teacher or parent reports of emotional competences of the children. Regarding the development of the MeKKi and the present study adding to the results of the pilot studies, the findings indicate much potential for this inventory. Overall, the present results confirm the results of the pilot studies of the MeKKi. Nonetheless, further improvements will help increase the quality of this inventory; for example, results indicate that items with differential item functioning should be revised or could be deleted.

The MeKKi could potentially find application in identifying deficits in emotional competence in children as young as preschool age, enabling the adoption of intervention or prevention programs. The correlation of the MeKKi and the well-established SDQ instrument in a pilot study seems promising even though it lacks statistical significance, which might be due to the limited sample size in the pilot studies. Further research should address the convergent and predictive validity with larger samples to investigate the association between psychopathology and the MeKKi subscales as well as other validated measures on emotional competence (which would have to be developed and evaluated in German). To establish the MeKKi as a suitable diagnostic instrument, further testing should also include clinical samples. This would make it possible to investigate the differential validity beyond the pilot studies. Furthermore, the MeKKi might be used to evaluate intervention programs fostering emotional competence. Overall, the results indicate that the MeKKi is very likely a suitable computer-based standardized inventory for assessing emotional competence on a multidimensional level in preschoolers and school-aged children.

## Summary

The results of our study indicate that the MeKKi is very likely a suitable computer-based standardized inventory for assessing emotional competence on a multidimensional level in preschoolers and school-aged children. The MeKKi was developed to assess five components of emotional competence: emotion vocabulary, emotion identification, emotion understanding, emotion expression, and emotion regulation. Results on acceptance ratings in participating children, validity, reliability, and factor structure provide support for the use of the MeKKi in research and clinical settings. The MeKKi could potentially find application in identifying deficits in emotional competence in children as young as preschool age, enabling the adoption of intervention or prevention programs.
